# Clinical characteristics of sarcoma patients: a population-based data analysis from a German clinical cancer registry

**DOI:** 10.1007/s00432-023-05350-5

**Published:** 2023-09-26

**Authors:** Jörg Andreas Müller, Karl-Stefan Delank, Kevin Laudner, Ian Wittenberg, Alexander Zeh, Dirk Vordermark, Daniel Medenwald

**Affiliations:** 1 Department of Radiation Oncology, University Hospital Halle (Saale), Ernst-Grube-Str. 40, 06120 Halle (Saale), Germany; 2 Institute of Medical Epidemiology, Biometry, and Informatics, Martin Luther University Halle-Wittenberg, Magdeburger Strasse 8, 06112 Halle (Saale), Germany; 3 Department of Orthopedics, Trauma, and Reconstructive Surgery, University Hospital Halle (Saale), Ernst-Grube-Str. 40, 06120 Halle (Saale), Germany; 4 Department of Health Sciences, Hybl Sports Medicine and Performance Center, University of Colorado Colorado Springs, 1420 Austin Bluffs, CO Colorado Springs, CO 80918 USA; 5 Clinical Cancer Registry Saxony-Anhalt (Klinische Krebsregister Sachsen-Anhalt GmbH), Doctor-Eisenbart-Ring 2, 39120 Magdeburg, Germany

**Keywords:** Soft tisse sarcoma, Cancer registry, Prognostic factors, Overall survival, Progression-free survival

## Abstract

**Purpose:**

Sarcomas are a heterogeneous group of malignant neoplasms with a wide range of histological types and occur in almost any anatomic site and side. This study evaluated the prognostic factors in sarcoma patients based on German clinical cancer registry data.

**Methods:**

The German clinical cancer register of Saxony-Anhalt was used for all data analyses. Sarcoma cases of all clinical or pathological T-stages (T1a–T4c), all N-stages (N0-3) and M-stages (0–1b) corresponding to the Union for International Cancer Control (UICC) stages I to IVB were considered. In our analyses, 787 cases diagnosed between 2005 and 2022 were included. Further, we assessed the association of cancer-related parameters with mortality and hazard ratios (HR) from the Cox proportional hazard models. We included sex, age at diagnosis, histological grade, T-, N- and M-stages, tumor size, tumor localization and tumor side as parameters in our regression models.

**Results:**

The majority of sarcoma patients were diagnosed with leiomyosarcoma (12%), liposarcoma (11%), angiosarcoma (5.3%) and myxofibrosarcoma (2.7%). In our univariate regression models, tumors localized in more than one location, head, face and neck region as well as the pelvis and lower extremity were associated with increased mortality risk (more than one location: HR 7.10, 95% CI 2.20–22.9; head, face and neck: HR 1.35, 95% CI 0.89–2.06; pelvis: HR 1.27, 95% CI 0.86–1.89; lower extremity: HR 1.44, 95% CI 1.05–1.96). Higher histological grades, UICC-grades and TNM-stages were related to a higher mortality risk. Differing histological subtypes had significant influence on overall survival and progression-free survival. Patients diagnosed with fibromyxoid sarcoma, rhabdomyosarcoma and angiosarcoma were related to higher mortality risk compared to other histological subtypes (fibromyxoid sarcoma: HR 5.2, 95% CI 0.71–38.1; rhabdomyosarcoma: HR 2.93, 95% CI 1.44–6.00; angiosarcoma: HR 1.07, 95% CI 0.53–2.18).

**Conclusions:**

Histological grade, tumor size, nodal and distant metastasis, tumor localization and histological subtype were determined as prognostic factors in terms of survival.

## Introduction

Sarcomas are a heterogeneous group of malignant neoplasms. These tumors arise from mesenchymal cells, which include dozens of histological types, and can occur in almost every anatomic site and side (Stiller et al. 2013). However, sarcoma is a rare disease with an annual incidence rate of 5.6 per 100,000 individuals in Europe (Stiller et al. 2013). Sarcomas account for over 20% of all pediatric solid malignant cancers, but less than 1% of all adult solid malignant cancers. The vast majority of diagnosed sarcomas are soft tissue sarcomas, while bone tumors account for just over 10% (Burningham et al. 2012).

Survival among sarcoma patients depends on a range of prognostic factors. Cancer survival rates have significantly improved over time, except among adolescent and young adult (AYA) (i.e., 15–39 years) (Tricoli et al. 2016). In 2021, a Japanese study investigated soft tissue sarcoma in AYA patients regarding risk factors for poor outcomes using a nationwide bone and soft tissue tumor registry in Japan. The results of the study showed that AYA age was not a prognostic factor for poor cancer survival among soft tissue sarcoma patients (Fukushima et al. 2021). In some publications, histological type and grading are highlighted as decisive prognostic factors (Gage et al. 2019; Maretty-Nielsen et al. 2014). Further prognostic factors for survival are initial metastases and performed surgery as major treatment-linked factors (Stoeckle et al. 2001). Gootee et al. investigated prognostic factors in leiomyosarcoma patients. They showed a significantly higher risk of mortality associated with older patients, tumors localized in the female reproductive organs, African-American patients, higher tumor stage, tumors treated with surgery alone without adjuvant radiation and tumors with positive microscopic, macroscopic, or indeterminate surgical margins (Gootee et al. 2020).

There are different therapeutic approaches for treatment of sarcoma patients. The primary treatment in early-stage sarcoma patients consists of a wide surgical resection to obtain tumor-free resection margins (Geer et al. 1992; Karakousis et al. 1991). Data from prospective studies support the use of radiotherapy (RT) in addition to surgery among appropriately selected patients in terms of an improvement in disease-free survival (DFS) with the exception of overall survival (OS) (Fleming et al. 1999; McKee et al. 2004; Pisters et al. 1996).

Preoperative chemoradiation has been shown to improve the prognosis of OS, DFS and local control rates in patients with stage II–III sarcomas located in the extremity and trunk region. However, one has to consider side effects such as acute reactions (Kraybill et al. 2010; Mullen et al. 2012).

Cassier et al. assessed the role of adjuvant RT following resection in liposarcoma patients. In this study, adjuvant RT following resection was associated with a reduction of local recurrence risk (Cassier et al. 2014). Another study aimed to determine whether the timing of RT has an effect on healing complications in soft tissue sarcoma of the limbs. Preoperative RT was found to be associated with a greater risk of healing complications while OS was slightly better compared with postoperative RT. The authors suggested that the decision for a specific of therapeutic regimen should depend on the timing of surgery and RT, as well as the size and anatomical site of the tumor (O'Sullivan et al. 2002).

Past guideline recommendations have often been based on case collections and meta-analysis. Hence, registry data analyses are needed to fill the gap in randomized controlled trials.

## Methods

### Data and materials

For the present study, data from the clinical cancer registry of Saxony-Anhalt were analyzed. This population-based registry is regulated by German federal and state law and incorporates data that are transferred from healthcare facilities in Saxony-Anhalt.

Among other information, data sets include structured information on tumor, node, metastasis (TNM)-stage, grading and histology, date of birth, cause and date of death, date of diagnosis (month as smallest temporal unit in each date variable) and treatment. Furthermore, information on treatment procedures such as administered radiation dose and fractionation or number of surgeries was included. Additionally, the TNM-stage referred to in this data set, to the clinical or pathological stage (if an operation was performed). Pathological stages were favored if both ratings differed. Some cases showed incomplete information. If information on subclassification of T-stages (e.g., T1a, T1b) was not available, cases were classified as subgroups T1–4. Likewise, Union for International Cancer Control (UICC)-stages were defined as I–IV. Analogous to former study designs, age groups were defined as follows: <  = 14 years, 15–29 years, 0–44 years, 45–59 years and older than 60 years (Fukushima et al. 2021; Xu et al. 2021).

Regarding a high number of histological subtypes, the cutoff for inclusion in the study was set at more than five documented cases for each subgroup. Furthermore, soft tissue sarcomas of the extremity and trunk not included in other subgroups were categorized as “soft tissue sarcoma.” Remaining cases were summarized as “other histological subtype.”

Tumor localization was defined based on International Statistical Classification of Diseases and Related Health Problems (ICD)-codes for sarcoma cases. All ICD-codes C49.0–C49.9 were used to assign tumor locations for all recorded sarcoma cases. We did not define tumor locations for organ-related sarcomas because of differing ICD-codes and classification criteria (e.g., pleural mesothelioma, mesothelioma, gastrointestinal stromal tumors). Further, tumor size was estimated by T-stage in accordance with the American Joint Committee on Cancer (AJCC) staging criteria (Cates 2018). Histological grades were defined as low, intermediate and high grade sarcoma according to the “Arbeitsgemeinschaft Deutscher Tumorzentren, ADT” (Stegmaier et al. 2019). For illustrative purposes, the 10 most frequent histological subtypes were illustrated with Kaplan–Meier curves.

All primary therapies within 365 days after diagnosis were considered in our analysis.

### Definition of periods

Cases diagnosed between 2005 and 2022 (most recent data with sufficient quality) were included for this analysis. Cases were censored at October 2022 (latest complete recording of death) or after 60 months to avoid a bias due to cases that died in more recent years, but whose changed survival status had not yet been considered in the data.

### Statistical analyses

We used proportional hazard Cox regression models to assess the association of cancer-related parameters with mortality and computed hazard ratios (HR) with 95% confidence intervals (CIs).

Furthermore, we computed univariate and multivariate Cox regression models. All models were adjusted for histological grade (as defined above), age at diagnosis, T-stage, N-stage, M-stage, tumor location based on ICD-codes as described above, histological subtype, tumor side, patient sex and UICC-stage. For illustrative purposes, Kaplan–Meier curves were created for all included risk factors. Our primary endpoints were overall survival (OS) and progression-free survival (PFS). Additionally, we computed median OS and PFS survival rates.

A significance level of 5% was used. All statistical analyses were performed using RStudio Version 1.4.1717 (RStudio 2020, Integrated Development for R. RStudio, PBC, Boston, MA, USA). All graphics were computed with the “gtsummary” package in RStudio (Sjoberg et al. 2021).

## Results

### Case selection

**Fig. 1:**
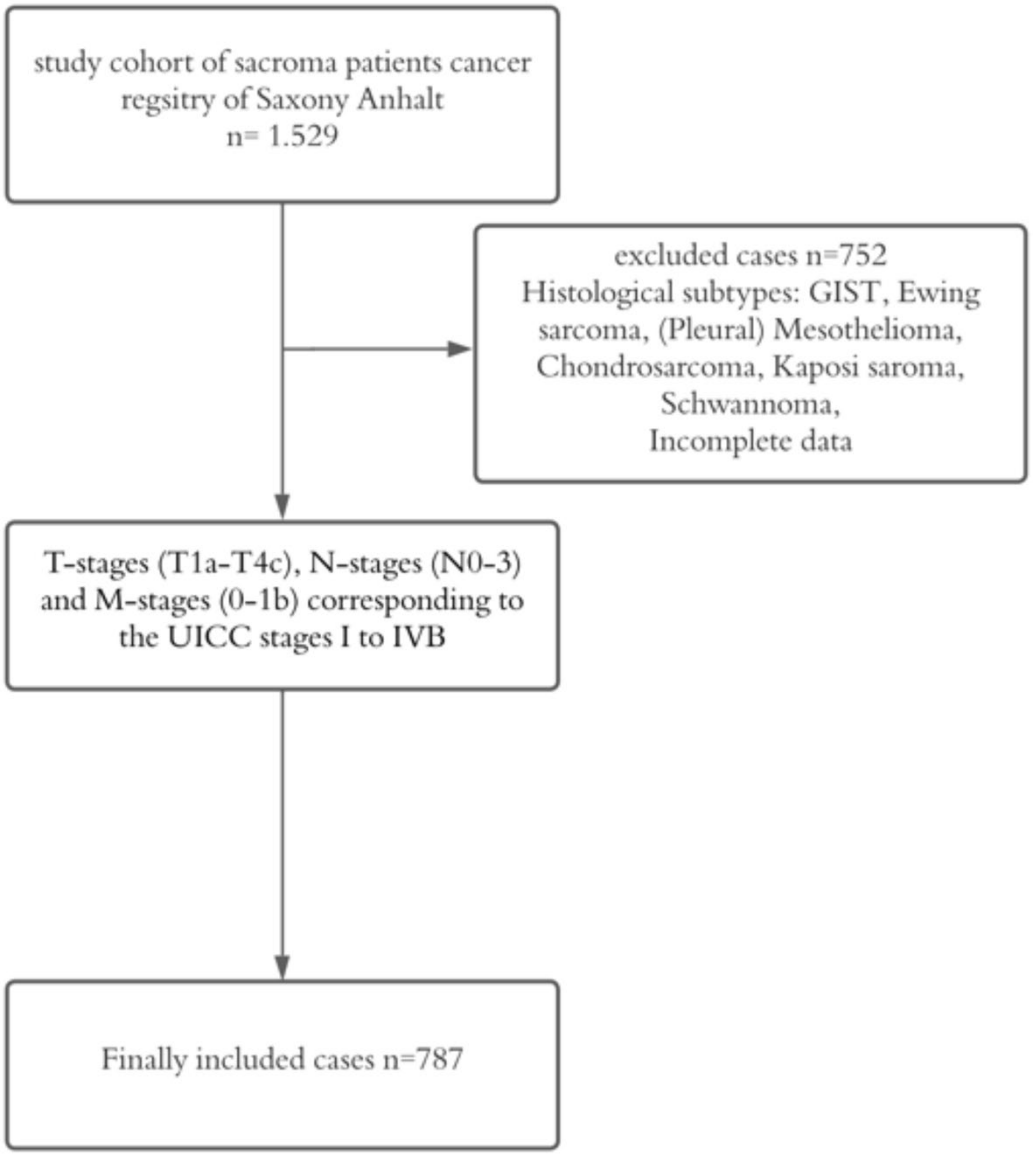
Flowchart of sample inclusion and exclusion criteria (GIST = gastrointestinal stromal tumor)

The total number of UICC stage I–VI sarcoma cases diagnosed between 2005 and 2022, provided by the cancer registry of Saxony-Anhalt, was 1529 (Cases were reported in accordance with the German Manual of Cancer Registration (Stegmaier et al. 2019)). In order to meet the specific aim of this study, we excluded subgroups with different tumor biology and low number of cases and focused on soft tissue sarcoma patients. Consequently, all patients diagnosed with gastrointestinal stromal tumors, Ewing sarcoma, (pleural) mesothelioma, chondrosarcoma, Kaposi sarcoma and schwannoma were excluded from further analysis. Incomplete data with undefined histological subtypes or lacking ICD-codes were removed from the study as well, leading to a total number of 752 excluded cases. This resulted in a total number of 787 cases included for analysis (Fig. 1).

### Patient characteristics

All patient characteristics can be viewed in Table 1. The majority of analyzed patients were men (838 male, female). Based on higher sarcoma prevalence rates in children and young adults, a subgroup analysis of patients younger than 40 years was performed, in accordance with former studies (Avila et al. 2018). 6.6% (*n* = 102) of included patients were younger than 40 years. On average, patients were 68 years old when they were diagnosed. The majority of sarcoma patients was diagnosed with leiomyosarcoma (12%), liposarcoma (11%), pleural mesothelioma (5.4%) and angiosarcoma (5.0%). In the younger age group, rhabdomyosarcoma (5.9%) and synovial sarcoma (5.9%) had a higher prevalence compared with patients > 40 years. One-third of all patients had a grade 3 (27%) or grade 4 (5.2%) sarcoma. In contrast, younger patients were more likely to have a low-grade sarcoma (G1: *n* = 11, 19%; G2: *n* = 16, 28%). In a high percentage of cases, information on histological grading was not determined (13%) or unknown (13%). The most frequent T-stages were stage 2 (15%) and 2b (18%). T-stages could be assessed in 34% percent of sarcoma cases. Furthermore, the vast majority of cases had no lymphonodal metastases (N-stage = N0, 63%). Likewise, 63% of the included cases were not diagnosed with distant metastases. Analogous to measured T-stages, N-stage data were missing in 32% of collected cases. M-status data could not be obtained in 11% of sarcoma cases. Twenty-seven percentage of the patients were classified as UICC-stage IV. Moreover, in the younger patient group, almost half of included cases were staged as UICC IV patients (*n* = 20, 44%). This high proportion of advanced cancer stages could be explained by the higher distant metastasis rate in younger patients (*n* = 17, 35%) compared to older patients (*n* = 152, 21%). Regarding tumor localization, the majority of sarcomas were located in the lower extremity (32%), abdomen (16%) and in the head, face and neck region (11%). The upper extremity region was affected in only 9.5% of analyzed cases. Younger patients had more sarcomas in the trunk (*n* = 8, 9.8%) and upper extremity (*n* = 12, 15%) region compared to patients older than 40 years. In contrast, thoracic sarcomas occurred more often in older patients (patients > 40 years: *n* = 132, 12%; patients < 40 years: *n* = 2, 2.4%). Concerning tumor side, left- and right-sided tumors were equally distributed (right side: *n* = 440, 45%; left side: *n* = 513, 52%). Midline and both-sided tumors were comparatively rare (midline: *n* = 24, 2.4%; both sides: *n* = 7, 0.7%). Most patients were treated with surgery (*n* = 484, 36%). Radiation therapy was performed after surgery in 287 (21%) cases. A large proportion of younger patients received additional systemic therapy after surgery and radiation therapy (*n* = 22, 26%). For not documented reasons, 15% of all included sarcoma patients did not receive any treatment (*n* = 201). However, in the younger patients’ group, 9 patients (10%) remained untreated.

**Table 1:** Patient characteristics

Characteristic	Overall, *N* = 1529^*1*^	< 40 years, *N* = 102^*1*^	> 40 years, *N* = 1427^*1*^
**Sex**			
Female	691 (45%)	51 (50%)	640 (45%)
Male	838 (55%)	51 (50%)	787 (55%)
**Age at Diagnosis**	68 (56, 77)	30 (23, 35)	69 (59, 77)
**Histological Subtype**			
Angiosarcoma	76 (5.0%)	1 (1.0%)	75 (5.3%)
Chondrosarcoma	17 (1.1%)	2 (2.0%)	15 (1.1%)
Dermatofibrosarcoma protuberans	10 (0.7%)	2 (2.0%)	8 (0.6%)
Fibromyxoid sarcoma	8 (0.5%)	1 (1.0%)	7 (0.5%)
Fibrosarcoma	23 (1.5%)	1 (1.0%)	22 (1.5%)
Gastrointestinal stromal tumor	50 (3.3%)	1 (1.0%)	49 (3.4%)
Kaposi sarcoma	11 (0.7%)	0 (0%)	11 (0.8%)
Leiomyosarcoma	186 (12%)	7 (6.9%)	179 (13%)
Liposarcoma	174 (11%)	12 (12%)	162 (11%)
Malignant peripheral nerve sheath tumor	30 (2.0%)	4 (3.9%)	26 (1.8%)
Mesothelioma	67 (4.4%)	1 (1.0%)	66 (4.6%)
Myxofibrosarcoma	42 (2.7%)	0 (0%)	42 (2.9%)
Other histological subtype	511 (33%)	40 (39%)	471 (33%)
Pleomorphic sarcoma	26 (1.7%)	1 (1.0%)	25 (1.8%)
Pleural mesothelioma	83 (5.4%)	1 (1.0%)	82 (5.7%)
Rhabdomyosarcoma	18 (1.2%)	6 (5.9%)	12 (0.8%)
Schwannoma	9 (0.6%)	2 (2.0%)	7 (0.5%)
Soft tissue sarcoma	140 (9.2%)	12 (12%)	128 (9.0%)
Spindle cell sarcoma	12 (0.8%)	1 (1.0%)	11 (0.8%)
Synovial sarcoma	23 (1.5%)	6 (5.9%)	17 (1.2%)
**Histological Grade**			
High grade	354 (23%)	15 (15%)	339 (24%)
Intermediate grade	1 (< 0.1%)	0 (0%)	1 (< 0.1%)
Low grade	350 (23%)	28 (27%)	322 (23%)
Unknown	824 (54%)	59 (58%)	765 (54%)
**T-Status**			
1	74 (8.8%)	4 (8.3%)	70 (8.9%)
1a	32 (3.8%)	5 (10%)	27 (3.4%)
1b	22 (2.6%)	0 (0%)	22 (2.8%)
2	125 (15%)	8 (17%)	117 (15%)
2a	36 (4.3%)	2 (4.2%)	34 (4.3%)
2b	152 (18%)	8 (17%)	144 (18%)
2c	1 (0.1%)	0 (0%)	1 (0.1%)
3	49 (5.8%)	3 (6.2%)	46 (5.8%)
3b	5 (0.6%)	0 (0%)	5 (0.6%)
4	44 (5.3%)	4 (8.3%)	40 (5.1%)
4a	6 (0.7%)	0 (0%)	6 (0.8%)
4b	5 (0.6%)	0 (0%)	5 (0.6%)
4c	1 (0.1%)	0 (0%)	1 (0.1%)
X	286 (34%)	14 (29%)	272 (34%)
Unknown	691 (45%)	54 (53%)	637 (44%)
**N-Status**			
0	479 (63%)	26 (58%)	453 (63%)
1	33 (4.3%)	7 (16%)	26 (3.6%)
1b	1 (0.1%)	0 (0%)	1 (0.1%)
2	3 (0.4%)	0 (0%)	3 (0.4%)
3	2 (0.3%)	0 (0%)	2 (0.3%)
X	241 (32%)	12 (27%)	229 (32%)
Unknown	770 (50%)	57 (56%)	713 (50%)
**M-Status**			
0	521 (67%)	29 (59%)	492 (67%)
1	169 (22%)	17 (35%)	152 (21%)
1a	1 (0.1%)	0 (0%)	1 (0.1%)
1b	1 (0.1%)	0 (0%)	1 (0.1%)
X	88 (11%)	3 (6.1%)	85 (12%)
Unknown	749 (49%)	53 (52%)	696 (49%)
**UICC-Status**			
I	20 (3.1%)	0 (0%)	20 (3.3%)
IA	38 (5.8%)	3 (6.7%)	35 (5.7%)
IB	88 (13%)	7 (16%)	81 (13%)
II	34 (5.2%)	2 (4.4%)	32 (5.2%)
IIA	18 (2.7%)	1 (2.2%)	17 (2.8%)
IIB	25 (3.8%)	2 (4.4%)	23 (3.8%)
III	54 (8.2%)	3 (6.7%)	51 (8.4%)
IIIA	40 (6.1%)	1 (2.2%)	39 (6.4%)
IIIB	37 (5.6%)	2 (4.4%)	35 (5.7%)
IV	179 (27%)	20 (44%)	159 (26%)
IVA	2 (0.3%)	0 (0%)	2 (0.3%)
IVB	8 (1.2%)	0 (0%)	8 (1.3%)
X	112 (17%)	4 (8.9%)	108 (18%)
Unknown	874 (57%)	57 (56%)	817 (57%)
**Therapy**			
Radiotherapy	36 (2.7%)	1 (1.2%)	35 (2.8%)
RT + Systemic Therapy	50 (3.7%)	5 (5.8%)	45 (3.5%)
Surgery	484 (36%)	25 (29%)	459 (36%)
Surgery + RT	287 (21%)	15 (17%)	272 (21%)
Surgery + RT + Systemic Therapy	188 (14%)	22 (26%)	166 (13%)
Systemic therapy	112 (8.2%)	9 (10%)	103 (8.1%)
Untreated	201 (15%)	9 (10%)	192 (15%)
Unknown	171 (11%)	16 (16%)	155 (11%)
**Tumor localization**			
Abdomen	193 (16%)	12 (15%)	181 (16%)
Head, Face and Neck	133 (11%)	9 (11%)	124 (11%)
Location unknown	57 (4.8%)	4 (4.9%)	53 (4.8%)
Lower extremity	381 (32%)	25 (30%)	356 (32%)
More than one location	8 (0.7%)	0 (0%)	8 (0.7%)
Pelvis	123 (10%)	10 (12%)	113 (10%)
Thorax	134 (11%)	2 (2.4%)	132 (12%)
Trunk	44 (3.7%)	8 (9.8%)	36 (3.3%)
Upper extremity	112 (9.5%)	12 (15%)	100 (9.1%)
Unknown	344 (22%)	20 (20%)	324 (23%)
**Tumor Side**			
Both sides	7 (0.7%)	1 (1.6%)	6 (0.7%)
Left side	513 (52%)	30 (48%)	483 (52%)
Midline	24 (2.4%)	1 (1.6%)	23 (2.5%)
Right side	440 (45%)	31 (49%)	409 (44%)
Unknown	545 (36%)	39 (38%)	506 (35%)
Tumor Size			
< = 5 cm	1,194 (78%)	82 (80%)	1,112 (78%)
> 10 cm and < 15 cm	15 (1.0%)	1 (1.0%)	14 (1.0%)
> 15 cm	12 (0.8%)	2 (2.0%)	10 (0.7%)
> 5 cm and < 10 cm	52 (3.4%)	5 (4.9%)	47 (3.3%)
Unknown	256 (17%)	12 (12%)	244 (17%)

^*1*^ *n* (%); Median (IQR)

### Survival analyses

Univariate and multivariate Cox regression models based on overall survival are viewed in Table 2. We computed univariate and multivariate Cox regression models for OS and PFS. In our univariate survival models, we could not find a survival benefit regarding sex. Female patients had a slightly higher mortality risk compared to men (HR 1.13, 95% CI 0.92–1.37). Younger age (15–29 years) was related to a worse survival compared to older patients > 60 years (HR 1.34, 95% CI 0.63–2.83). Sarcomas identified in more than one location had a higher mortality risk compared to sarcomas located at a single site (HR 7.10, 95% CI 2.20–22.9). Upper extremity sarcomas were associated with a better overall survival (HR 0.84, 95% CI 0.55–1.26) compared to lower extremity sarcomas (HR 1.44, 95% CI 1.05–1.96). Patients diagnosed with pelvic and thoracic sarcomas had a slightly higher mortality risk (Pelvis: HR 1.27, 95% CI 0.86–1.89; Thorax: HR 1.24, 95% CI 0.84–1.82) compared to patients with sarcomas localized in the head and neck or trunk region (Head, Face and Neck: HR 1.35, 95% CI 0.89–2.06; Trunk: HR: 1.10, 95% CI 0.58–2.09).

**Table 2:** Univariate and multivariate Cox regression models based on overall survival

Characteristic	Univariable				Multivariable		
	*N*	HR^*1*^	95% CI^*1*^	*p*-value	HR^*1*^	95% CI^*1*^	*p*-value
**Sex**	407			0.2			
Male		–	–		–	–	
Female		1.13	0.92, 1.37		1.59	0.80, 3.16	0.2
**Age Groups**	407			0.2			
> 60		–	–		–	–	
15–29		1.34	0.63, 2.83		0.08	0.00, 5.44	0.2
30–44		0.92	0.58, 1.44		0.23	0.02, 2.28	0.2
45–59		0.79	0.61, 1.02		0.27	0.10, 0.71	0.008
**Tumor Localization**	363			< 0.001			
Abdomen		–	–		–	–	
Head, face and neck		1.35	0.89, 2.06		2.05	0.40, 10.4	0.4
Location unknown		3.06	1.81, 5.20				
Lower extremity		1.44	1.05, 1.96		1.47	0.55, 3.88	0.4
More than one location		7.10	2.20, 22.9		3.74	0.18, N.C.	0.4
Pelvis		1.27	0.86, 1.89		0.70	0.20, 2.49	0.6
Thorax		1.24	0.84, 1.82		0.58	0.13, 2.57	0.5
Trunk		1.10	0.58, 2.09		0.46	0.09, 2.33	0.3
Upper extremity		0.84	0.55, 1.26		0.95	0.27, 3.32	> 0.9
**Histological grade**	407			< 0.001			
High grade		–	–		–	–	
Low grade		0.48	0.37, 0.63		0.44	0.19, 0.99	0.047
Unknown		0.85	0.67, 1.07		0.91	0.37, 2.24	0.8
**Histological Subtype**	407			0.001			
Angiosarcoma		1.07	0.53, 2.18		9.76	0.86, N.C.	0.065
Dermatofibrosarcoma protuberans		1.24	0.17, 8.97				
Fibromyxoid sarcoma		5.20	0.71, 38.1		18.0	1.13, N.C.	0.041
Fibrosarcoma		0.61	0.34, 1.10		4.00	0.83, 19.4	0.085
Leiomyosarcoma		0.59	0.42, 0.82		1.38	0.46, 4.18	0.6
Liposarcoma		0.55	0.39, 0.79		0.81	0.27, 2.47	0.7
Malignant peripheral nerve sheath tumor		0.82	0.47, 1.44		24.9	0.78, 792	0.068
Myxofibrosarcoma		0.42	0.23, 0.79		0.86	0.18, 4.07	0.9
Pleomorphic sarcoma		0.78	0.43, 1.41		0.64	0.10, 4.27	0.6
Rhabdomyosarcoma		2.93	1.44, 6.00		21.6	0.79, N.C.	0.068
Soft tissue sarcoma		0.74	0.52, 1.05		1.46	0.49, 4.33	0.5
Spindle cell sarcoma		0.56	0.24, 1.31		1.71	0.16, 18.4	0.7
Synovial sarcoma		0.94	0.48, 1.86		4.14	0.90, 19.1	0.069
**Tumor Side**	277			0.4			
Both sides		–	–				
Left Side		1.12	0.28, 4.55		–	–	
Midline		2.48	0.22, 27.6				
Right side		1.36	0.33, 5.50		0.92	0.47, 1.80	0.8
**Tumor Size**	407			0.041			
< = 5 cm		–	–		–	–	
> 10 cm and < 15 cm		7.47	1.03, 54.0				
> 15 cm		5.95	1.47, 24.1				
> 5 cm and < 10 cm		1.41	0.82, 2.40		1.84	0.42, 8.13	0.4
unknown		1.44	0.97, 2.16		0.67	0.15, 3.00	0.6
**T-Stage**	225			0.010			
1		–	–		–	–	
2		1.20	0.66, 2.19		0.28	0.05, 1.53	0.14
3		2.81	1.23, 6.43		2.01	0.14, 28.0	0.6
4		2.54	1.18, 5.48		4.70	0.22, 103	0.3
Unknown		1.55	0.84, 2.86		0.26	0.04, 1.83	0.2
**N-Stage**	212			0.006			
0		–	–		–	–	
1		3.39	1.76, 6.54		1.34	0.31, 5.77	0.7
1b		11.4	1.52, 84.7				
X		1.10	0.82, 1.48		1.29	0.51, 3.27	0.6
**M-Stage**	219			< 0.001			
0		–	–		–	–	
1		1.68	1.25, 2.26		0.12	0.00, 6.05	0.3
1a		6.04	0.83, 44.2		156	9.09, N.C.	< 0.001
X		0.49	0.30, 0.81		1.08	0.30, 3.90	> 0.9
**UICC-Stage**	195			0.004			
I		–	–		–	–	
IA		3.25	1.05, 10.1		1.76	0.15, 20.1	0.7
IB		1.46	0.56, 3.79		3.12	0.66, 14.8	0.2
II		1.84	0.53, 6.39		1.19	0.24, 5.90	0.8
IIA		1.81	0.48, 6.76		63.8	4.31, N.C.	0.003
IIB		1.63	0.54, 4.87		2.10	0.34, 12.9	0.4
III		2.76	1.04, 7.29		5.17	1.01, 26.3	0.048
IIIA		5.17	1.61, 16.6		8.39	1.24, 56.7	0.029
IIIB		3.85	1.28, 11.6		0.61	0.04, 8.66	0.7
IV		3.38	1.36, 8.43		41.5	1.08, N.C.	0.046
IVA		13.7	1.56, 121				
IVB		3.47	0.67, 18.0				
X		2.11	0.81, 5.55		3.27	0.53, 20.3	0.2

^*1*^*HR*  Hazard Ratio, *CI* Confidence Interval, *N.C.* not converged

Regarding adjusted covariates, lower UICC-stages were associated with a longer survival in our univariate Cox regression models. Further, higher histological grades and TNM-stages were related to a higher mortality risk. In our regression models, tumor side had no significant influence on overall survival. Left- and right-sided tumors had a statistically non-significant worse survival outcome related to both-sided sarcomas (left side: HR 1.12, 95% CI 0.28–4.55; right side: HR 1.36, 95% CI 0.33–5.50). Moreover, differing histological subtypes had significant influence on overall survival. Patients diagnosed with fibromyxoid sarcoma, rhabdomyosarcoma and angiosarcoma were related to higher mortality risk compared to other histological subtypes (fibromyxoid sarcoma: HR 5.2, 95% CI 0.71–38.1; rhabdomyosarcoma: HR 2.93, 95% CI 1.44–6.00; angiosarcoma: HR 1.07, 95% CI 0.53–2.18). In our data, liposarcoma, leiomyosarcoma and myxofibrosarcoma diagnosis were beneficial in terms of survival (liposarcoma: HR 0.55, 95% CI 0.39–0.79; leiomyosarcoma: HR 0.59, 95% CI 0.42–0.82; myxofibrosarcoma: HR 0.42, 95% CI 0.23–0.79) (see Table 2).

**Fig. 2:**
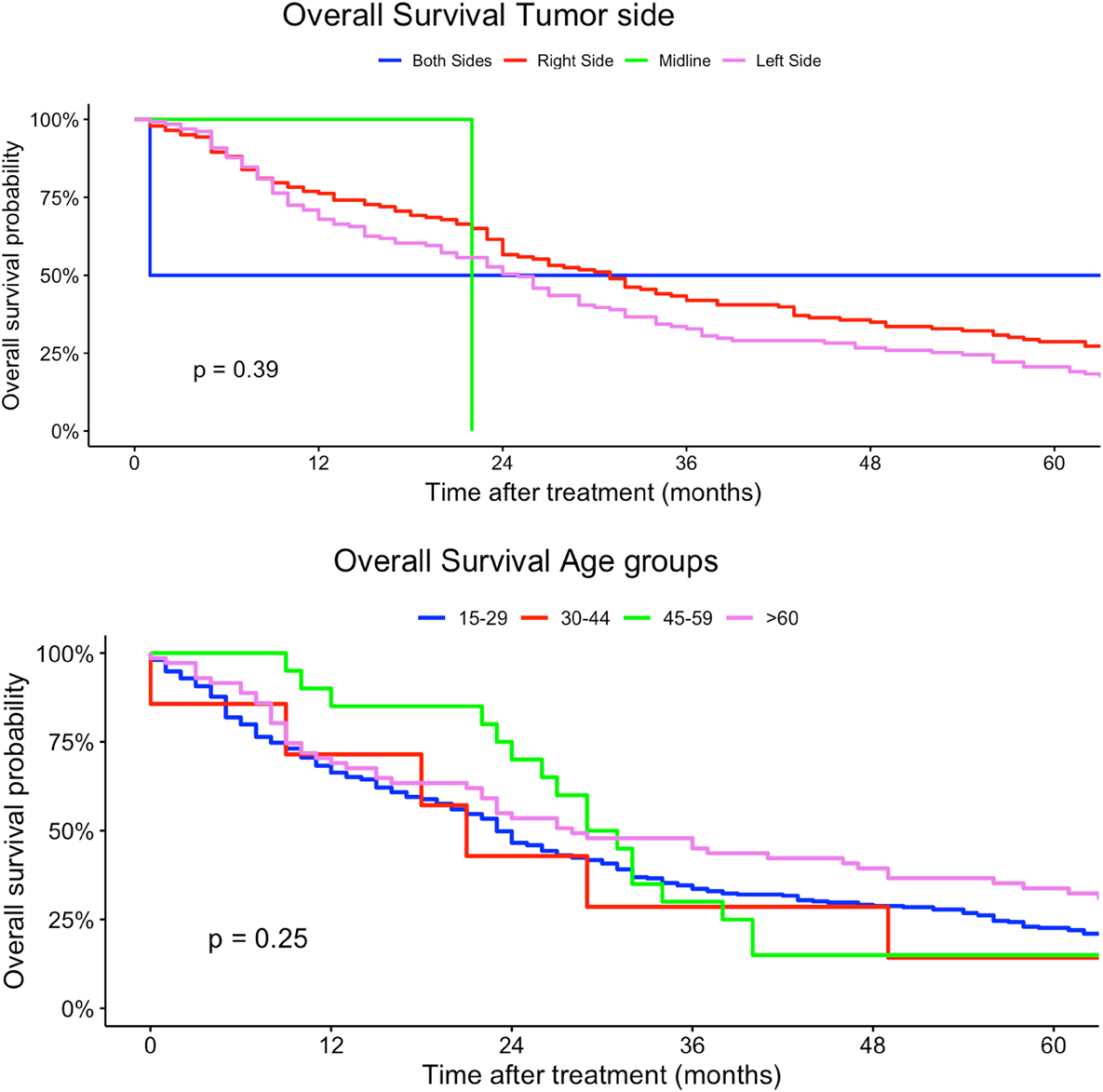
Kaplan-Meier curves of all patients (outcome: overall survival). The graph above is based on tumor side. Right-sided tumors were compared to left-sided, midline and both sided tumors. The bottom curve shows survival probabilities based on age groups. All graphs were censored 60 months after treatment

**Fig. 3:**
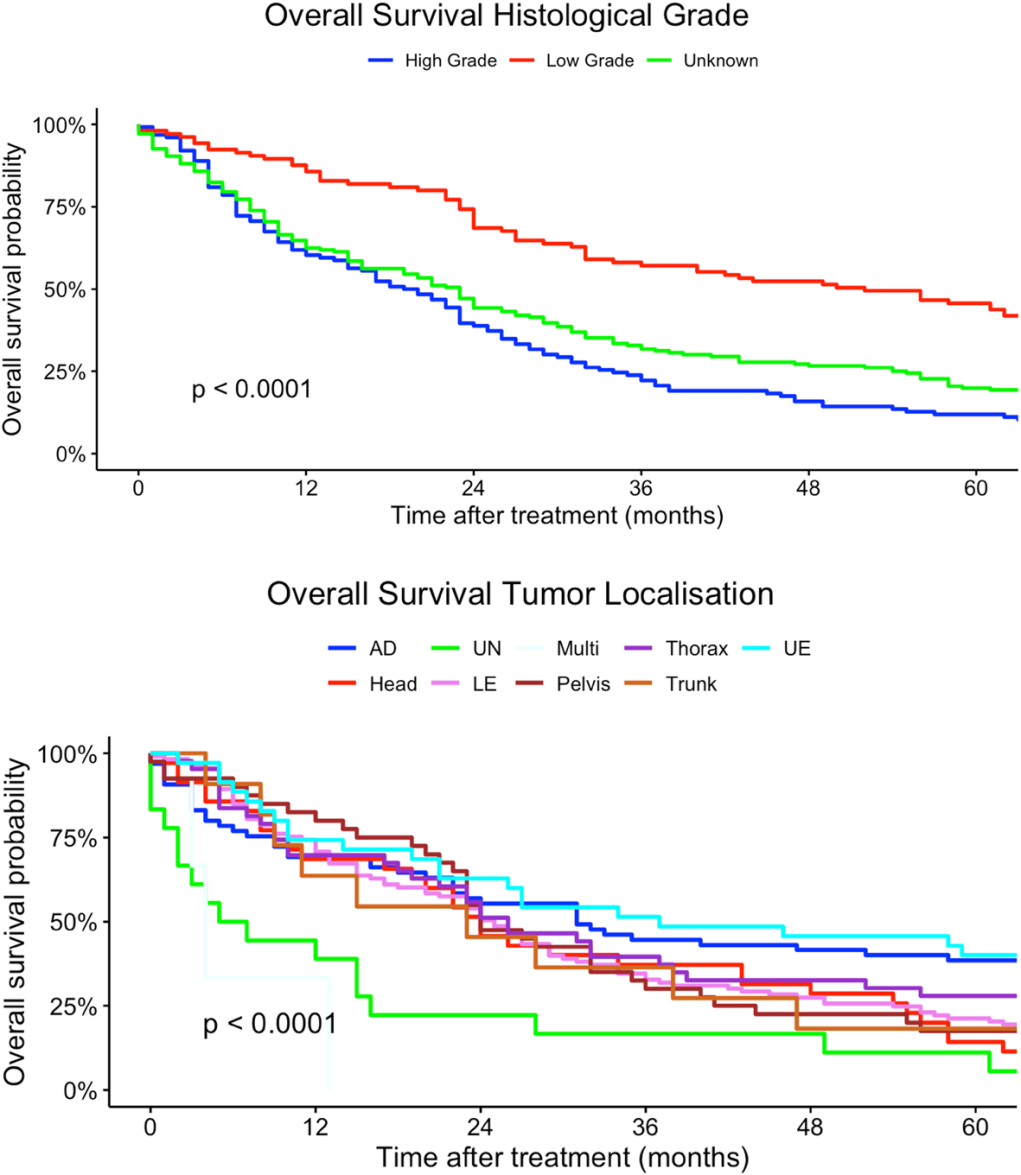
Kaplan–Meier curves of all patients (outcome: overall survival). The upper graph shows histological grades related to overall survival probability. The lower illustration shows survival based on tumor localization (UN = location unknown, Multi = more than one location, UE = upper extremity, LE = lower extremity, Head = head, face and neck). All graphs were censored 60 months after treatment

**Fig. 4:**
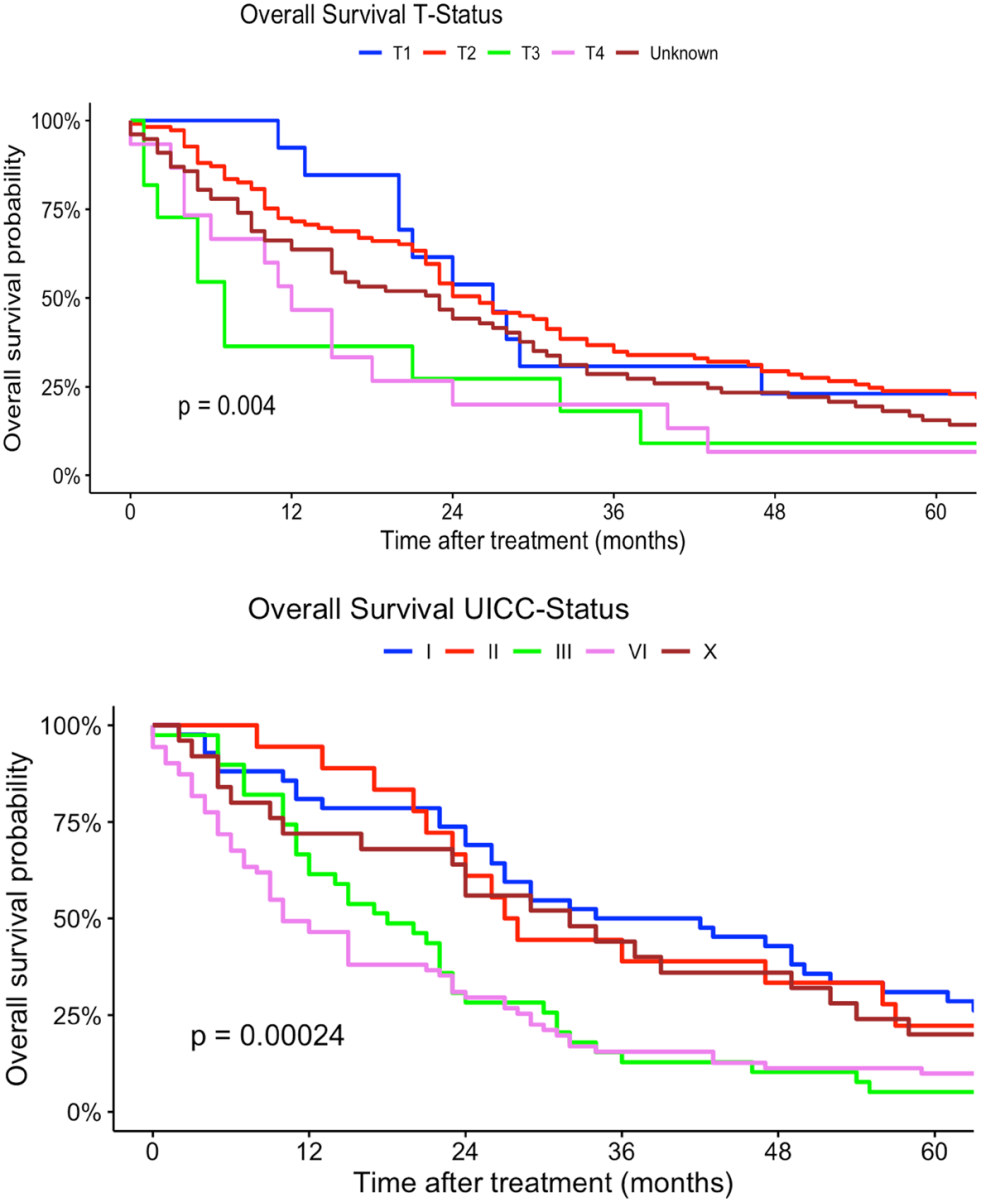
Kaplan–Meier curves of all patients (outcome: overall survival). The upper graph shows T-stages (Unknown = Tumor size could not be measured). UICC-stages are shown on the graph below (X = UICC stage could not be measured). All graphs were censored 60 months after treatment

**Fig. 5:**
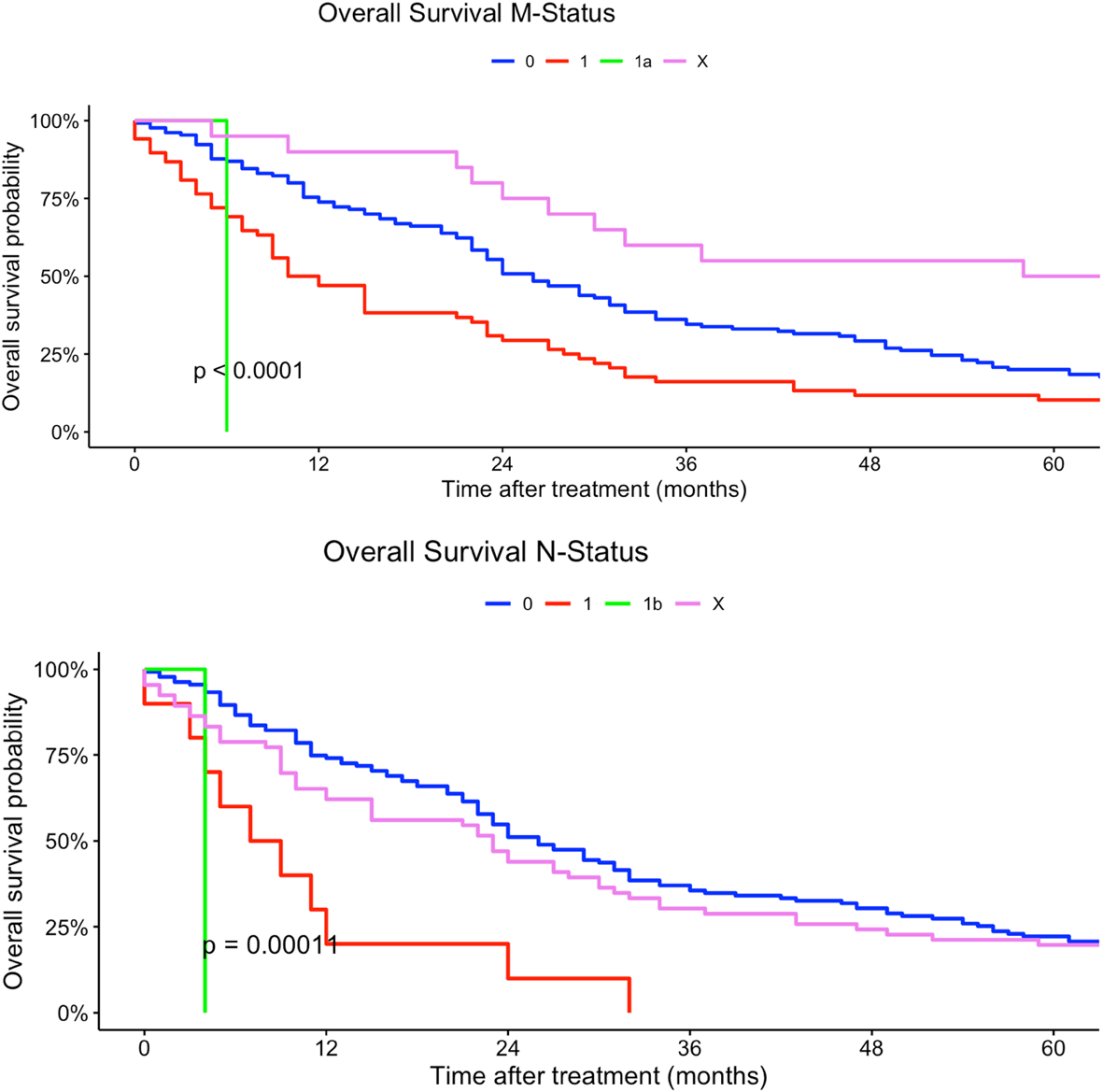
Kaplan–Meier curves of all patients (outcome: overall survival). The upper curve shows M-stages (X = Distant metastasis could not be measured). The lower graph deals with N-stages (X = Nodal metastases could not be measured). All graphs were censored 60 months after treatment

**Fig. 6:**
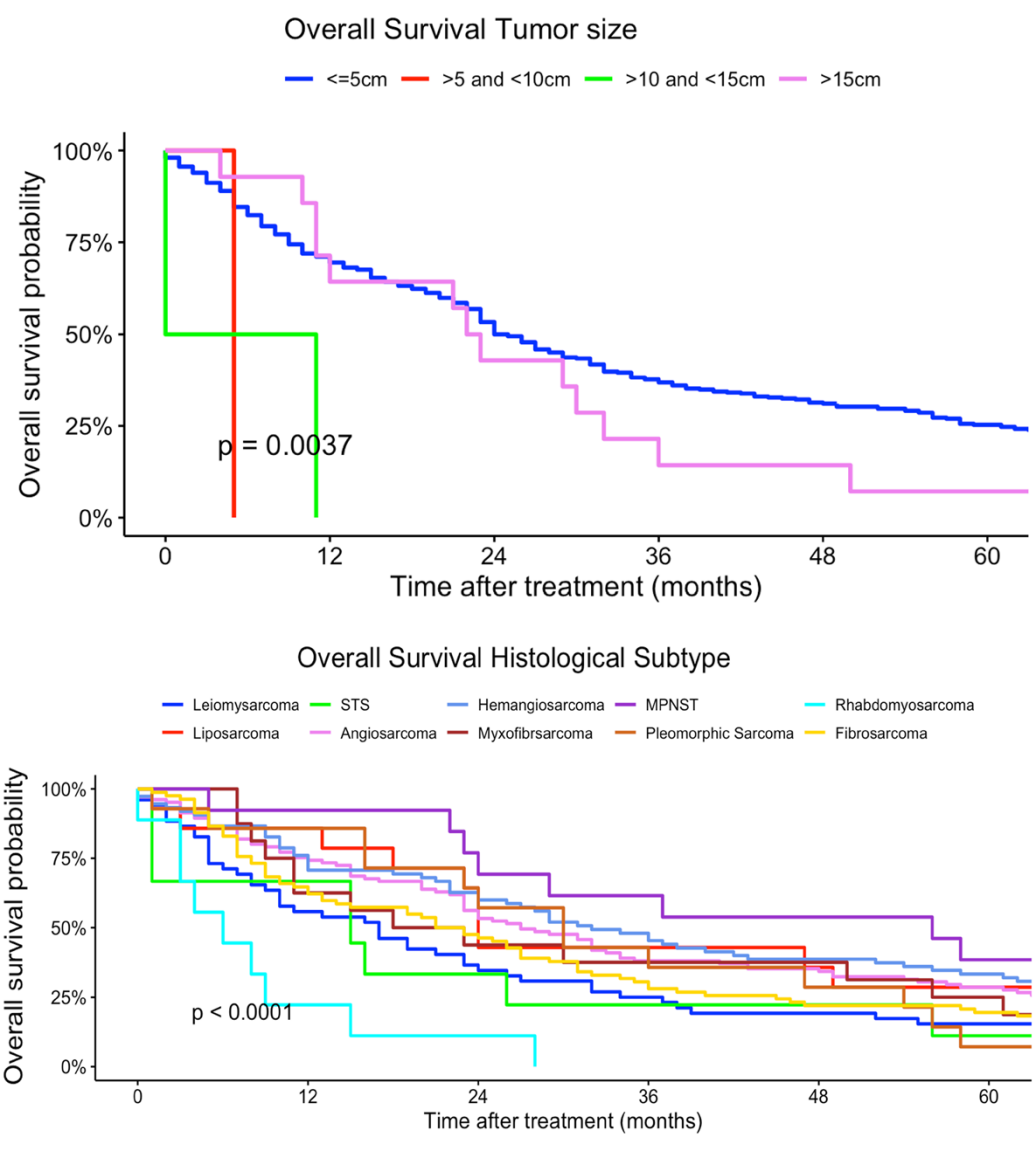
Kaplan–Meier curves of all patients (outcome: overall survival). The curve above shows tumor size. The graph below represents with histological subtypes (STS = soft tissue sarcoma, MPNST = malignant peripheral nerve sheath tumor). All graphs were censored 60 months after treatment

In our multivariate regression models, female patients had a higher mortality risk compared to men (HR 1.13, 95% CI 0.92–1.37). Differing to our univariate regression models, a younger age at diagnosis was not a strong risk factor in terms of survival compared with patients > 60 years (30–44 years: HR 0.23, 95% CI 0.02–2.28). In contrast, tumor localization had no statistically significant effect on survival. Multilocular tumor localizations, tumors localized in head, face and neck region as well as lower extremity sarcomas were associated with increased mortality risk (head, face and neck: HR 2.05, 95% CI 0.40–10.4; more than one location: HR 3.74, 95% CI 0.18–79.9; lower extremity: HR 1.47, 95% CI 0.55–3.88). Similar to our univariate regression results, worse histological grades and higher TNM-stages (T-status) were related to a higher mortality risk. Nodal and organ-related metastases were associated with higher mortality. Lower UICC-stages were associated with a better survival in our multivariate Cox regression models. Right-sided tumors had a slightly better survival compared to both-sided tumors (HR 0.92, 95% CI 0.47–1.80). Angiosarcoma, fibromyxoid sarcoma, synovial sarcoma and rhabdomyosarcoma were related to a higher mortality risk compared to other histological subtypes (angiosarcoma: HR 9.76, 95% CI 0.86–110; rhabdomyosarcoma: HR 21.6, 95% CI 0.79–591; fibromyxoid sarcoma: HR 5.20, 95% CI 1.13–287; synovial sarcoma: HR 4.14, 95% CI 0.90–19.1). In comparison, liposarcoma and pleomorphic sarcoma and myxofibrosarcoma patients had a survival benefit related to other subtypes (liposarcoma: HR 0.81, 95% CI 0.27–2.47; pleomorphic sarcoma: HR 0.64, 95% CI 0.10–4.27; HR myxofibrosarcoma: 0.86, 95% CI 0.18–4.07) (Table 2). Figures 2, 3, 4, 5, 6 show Kaplan–Meier curves of all included prognostic factors based on overall survival.

The median overall survival (OS) of all patients was 24 months (95% CI 16–22 months). Median survival rates differed between histological subtypes. Myxofibrosarcoma, spindle cell sarcoma, liposarcoma and leiomyosarcoma had the best median survival (myxofibrosarcoma: 56 months; spindle cell sarcoma: 50 months; liposarcoma: 32 months; Leiomyosarcoma: 27 months). Fibromyxoid sarcoma and rhabdomyosarcoma had the worst median survival rates (fibromyxoid sarcoma: 5 months; rhabdomyosarcoma: 6 months) (Table 3).

**Table 3:** Median overall and progression-free survival of histological subtypes

	N	Median OS (95% CI) (months)	N	Median PFS (95% CI) (months)
	407		214	
Angiosarcoma		17 (10, 26)		7.0 (5.0, 15)
Dermatofibrosarcoma protuberans		22 (—, —)		1.0 (—, —)
Fibromyxoid sarcoma		5.0 (—, —)		—
Fibrosarcoma		24 (23, 115)		22 (18, —)
Leiomyosarcoma		27 (23, 34)		25 (15, 36)
Liposarcoma		32 (24, 54)		18 (12, 36)
Malignant peripheral nerve sheath tumor		20 (11, 70)		9.0 (7.0, —)
Myxofibrosarcoma		56 (24, —)		40 (7.0, —)
Pleomorphic sarcoma		30 (23, 58)		21 (17, —)
Rhabdomyosarcoma		6.0 (3.0, —)		2.5 (2.0, —)
Soft tissue sarcoma		22 (14, 29)		9.0 (6.0, 21)
Spindle cell sarcoma		50 (22, —)		–
Synovial sarcoma		18 (8.0, —)		–

In addition, median progression-free survival was computed. The median progression-free survival was 15 months (95% CI 12–19 months). The histological subtypes with the best median non-progression rates were myxofibrosarcoma, leiomyosarcoma and fibrosarcoma (myxofibrosarcoma: 40 months; leiomyosarcoma: 25 months; fibrosarcoma: 22 months). Rhabdomyosarcoma, angiosarcoma and MPNST were the histological subtypes with the earliest progression rates (rhabdomyosarcoma: 2.5 months; MPNST: 9 months; angiosarcoma: 7 months). All extremity and trunk soft tissue sarcomas also had an early median progression at 9 months (95% CI 6–21 months).

Regarding our second endpoint PFS (Table 4), women had a progression-free survival benefit in the univariate regression model. The multivariate model showed an association of female sex and worse survival (univariate model female: HR 0.96, 95% CI 0.73–1.27; multivariate model female: HR 1.15, 95% CI 0.42–3.17).

**Table 4:** Univariate and multivariate Cox regression models based on PFS

Characteristic	Univariable				Multivariable		
	*N*	HR^*1*^	95% CI^*1*^	*p*-value	HR^*1*^	95% CI^*1*^	*p*-value
**Sex**	214			0.8			
Male		–	–		–	–	
Female		0.96	0.73, 1.27		1.15	0.42, 3.17	0.8
**Age Groups**	214			0.11			
> 60		–	–		–	–	
15–29		1.21	0.59, 2.48		60.5	2.51, N.C.	0.011
30–44		1.14	0.66, 1.98		17.3	1.24, N.C.	0.034
45–59		0.70	0.51, 0.97		1.57	0.42, 5.90	0.5
**Tumor Localization**	181			0.5			
Abdomen		–	–		–	–	
Head, face and neck		1.51	0.75, 3.02		1.60	0.14, 18.8	0.7
Location unknown		1.12	0.49, 2.55		2.39	0.28, 20.7	0.4
Lower extremity		1.37	0.86, 2.16		0.32	0.09, 1.10	0.072
More than one location		3.67	0.49, 27.3		0.37	0.01, 9.91	0.6
Pelvis		0.93	0.54, 1.59		3.55	0.69, 18.1	0.13
Thorax		0.96	0.57, 1.62		0.33	0.06, 1.90	0.2
Trunk		1.42	0.55, 3.70		0.56	0.08, 3.98	0.6
Upper extremity		0.83	0.49, 1.40		0.23	0.05, 1.06	0.059
**Histological Grade**	214			0.032			
High grade		–	–		–	–	
Low grade		0.62	0.42, 0.89		0.94	0.28, 3.20	> 0.9
Unknown		0.73	0.54, 1.01		1.51	0.36, 6.41	0.6
**Histological Subtype**	214			0.002			
Angiosarcoma		1.06	0.41, 2.78		0.97	0.10, 9.91	> 0.9
Dermatofibrosarcoma protuberans		30.6	3.60, 260				
Fibromyxoid sarcoma		0.81	0.11, 6.00		0.50	0.03, 9.78	0.6
Fibrosarcoma		0.49	0.23, 1.05				
Leiomyosarcoma		0.48	0.29, 0.79		0.32	0.07, 1.51	0.15
Liposarcoma		0.45	0.27, 0.73		0.69	0.17, 2.75	0.6
Malignant peripheral nerve sheath tumor		0.72	0.37, 1.40		0.27	0.02, 3.24	0.3
Myxofibrosarcoma		0.29	0.14, 0.64		1.03	0.10, 10.9	> 0.9
Pleomorphic sarcoma		0.70	0.21, 2.34		0.44	0.04, 5.52	0.5
Rhabdomyosarcoma		8.96	1.99, 40.3				
Soft tissue sarcoma		0.71	0.43, 1.15		1.85	0.38, 9.05	0.4
Synovial sarcoma		0.60	0.29, 1.24		0.11	0.01, 1.52	0.10
**Tumor Side**	146			0.3			
Left side		–	–		–	–	
Midline		2.45	0.60, 10.1		1.06	0.04, 31.2	> 0.9
Right side		1.25	0.90, 1.75		1.01	0.38, 2.71	> 0.9
**Tumor Size**	214			> 0.9			
< = 5 cm		–	–		–	–	
> 15 cm		0.66	0.09, 4.72				
> 5 cm and < 10 cm		1.01	0.55, 1.85		1.52	0.28, 8.35	0.6
unknown		1.13	0.71, 1.80		0.99	0.11, 9.38	> 0.9
**T-Stage**	118			0.2			
1		–	–		–	–	
2		2.16	0.85, 5.45		6.95	0.17, 291	0.3
3		1.91	0.36, 10.1		112	0.76, N.C.	0.064
4		3.93	1.25, 12.4		248	2.57, N.C.	0.018
Unknown		1.87	0.73, 4.81		4.77	0.05, N.C.	0.5
**N-Stage**	118			0.4			
0		–	–		–	–	
1		1.78	0.72, 4.40		5.17	0.45, 59.0	0.2
X		0.88	0.56, 1.37		0.74	0.20, 2.69	0.6
**M-Stage**	122			0.013			
0		–	–		–	–	
1		1.41	0.88, 2.25		7.94	0.99, 63.4	0.051
X		0.46	0.23, 0.92		1.12	0.10, 12.2	> 0.9
**UICC-Stage**	107			0.093			
I		–	–		–	–	
IA		1.45	0.42, 5.06		178	0.52, N.C.	0.082
IB		1.85	0.64, 5.36		13.8	0.83, 227	0.067
II		8.38	0.94, 74.7		146	1.56, N.C.	0.031
IIA		1.06	0.30, 3.75		44.5	1.26, N.C.	0.037
IIB		4.37	1.32, 14.5		72.8	3.42, N.C.	0.006
III		2.09	0.77, 5.70		12.8	1.37, N.C.	0.025
IIIA		3.00	0.93, 9.72		16.9	1.14, N.C.	0.040
IIIB		3.20	0.97, 10.6				
IV		2.77	1.05, 7.34				
X		1.35	0.50, 3.63		4.13	0.34, 49.8	0.3

^*1*^*HR* Hazard Ratio, *CI*  Confidence Interval, *N.C.* not converged

**Fig. 7:**
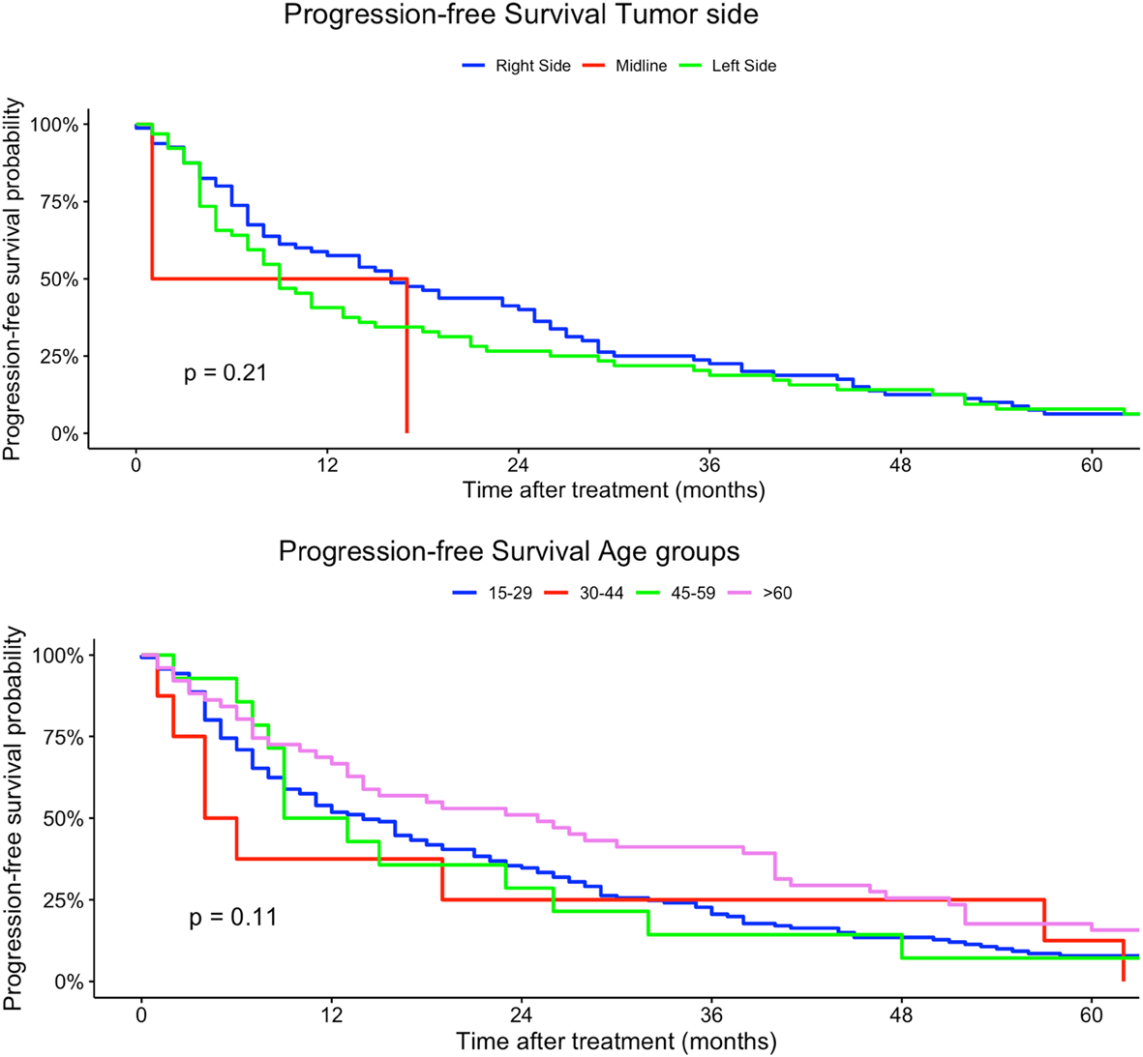
Kaplan–Meier curves of all patients (outcome: progression-free survival). The graph above is based on tumor side. Right-sided tumors were compared to left-sided, midline and both-sided tumors. The bottom curve shows progression-free survival probabilities based on age groups. All graphs were censored 60 months after treatment

**Fig. 8:**
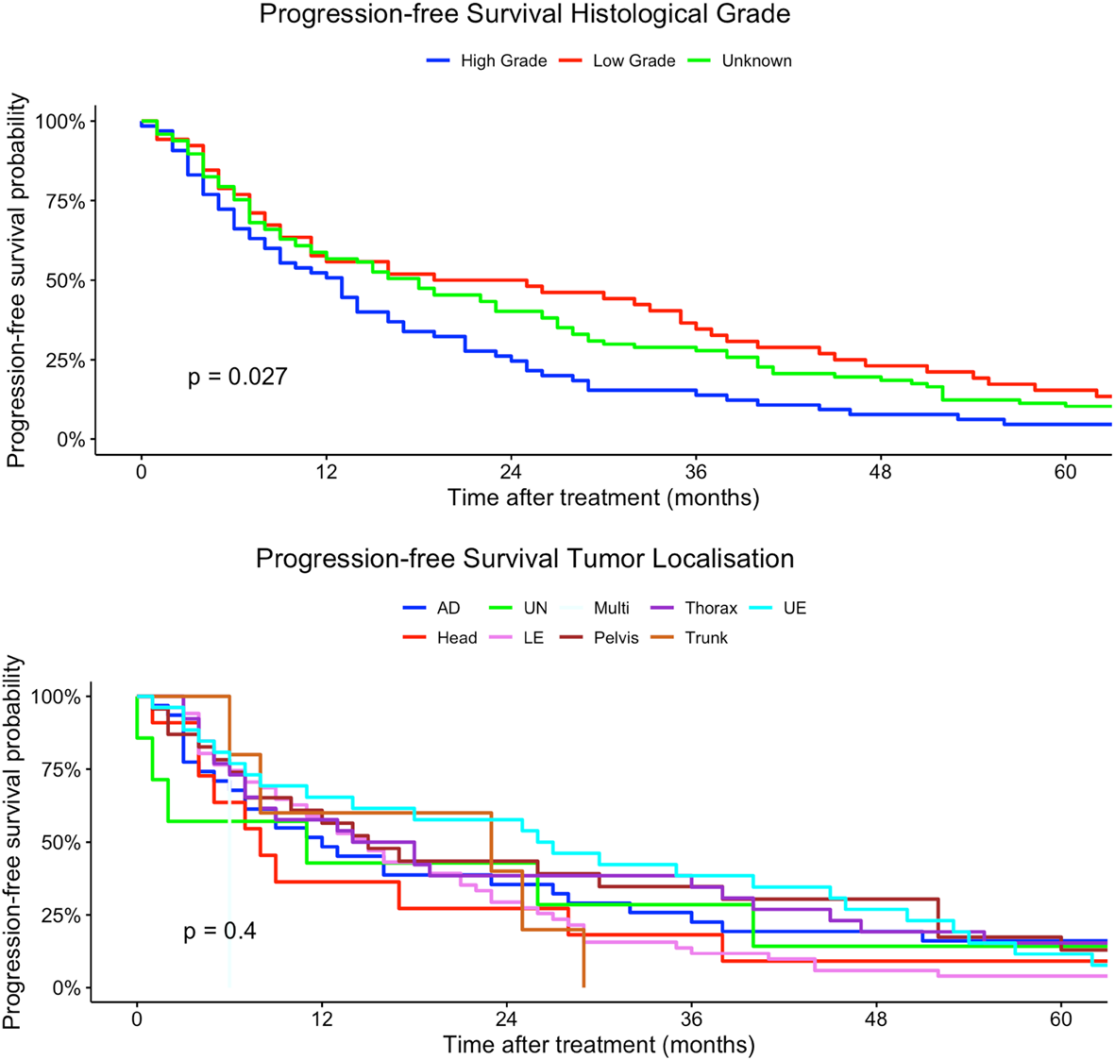
Kaplan–Meier curves of all patients (outcome: progression-free survival). The upper graph shows histological grades related to overall survival probability. The lower diagram shows PFS based on tumor localization (UN = location unknown, Multi = more than one location, UE = upper extremity, LE = lower extremity, Head = head, face and neck). All graphs were censored 60 months after treatment

**Fig. 9:**
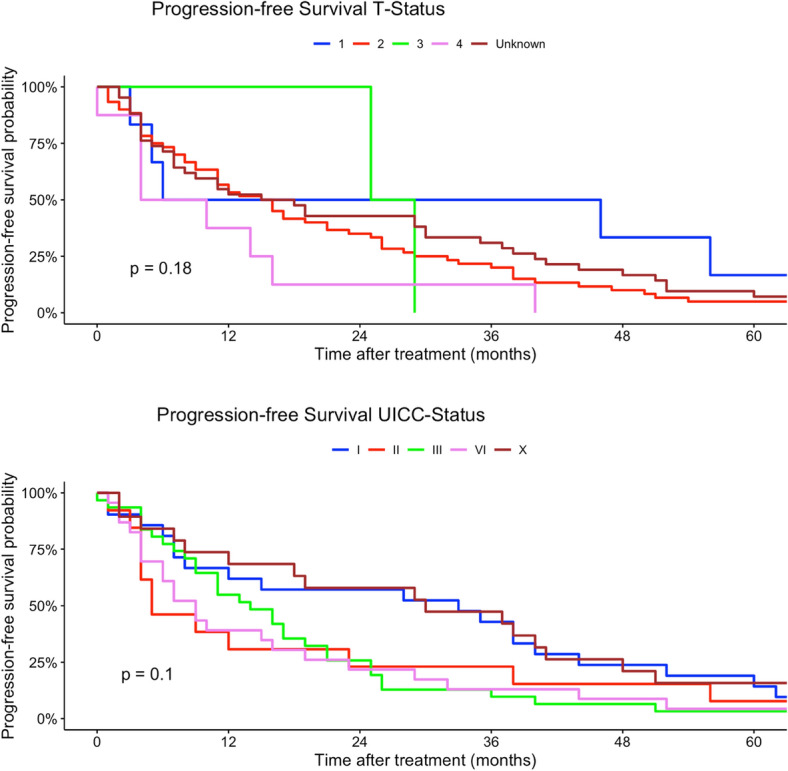
Kaplan–Meier curves of all patients (outcome: progression-free survival). The upper graph shows T-stages (Unknown = main tumor size could not be measured). UICC-stages are shown on the graph below (X = UICC stage could not be measured). All graphs were censored 60 months after treatment

**Fig. 10:**
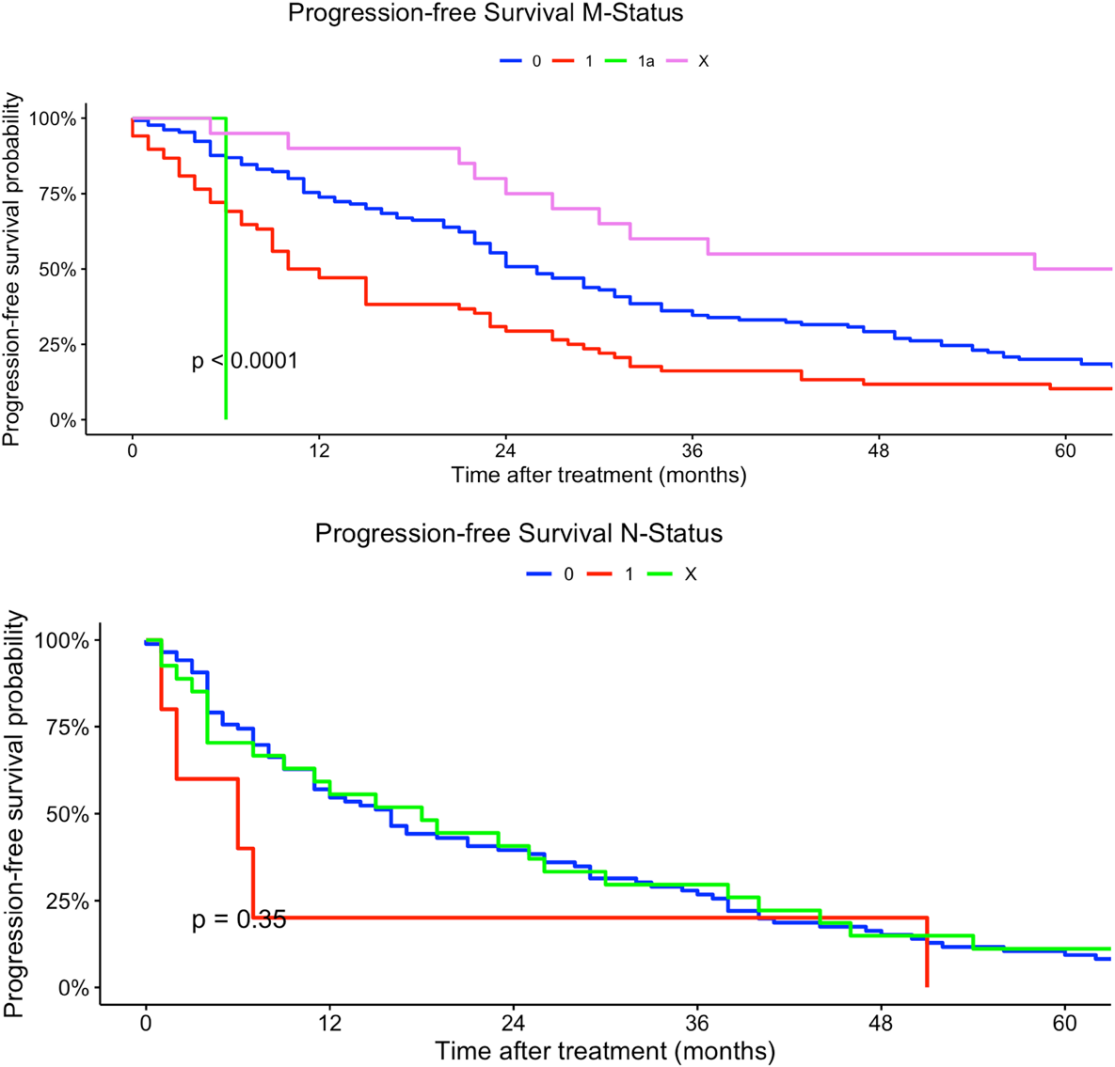
Kaplan–Meier curves of all patients (outcome: progression-free survival). The upper curve shows M-stages (X = Distant metastasis could not be measured). The lower graph visualizes the association between progression-free survival and N-stages (X = Nodal metastasis could not be measured). All graphs were censored 60 months after treatment

**Fig. 11:**
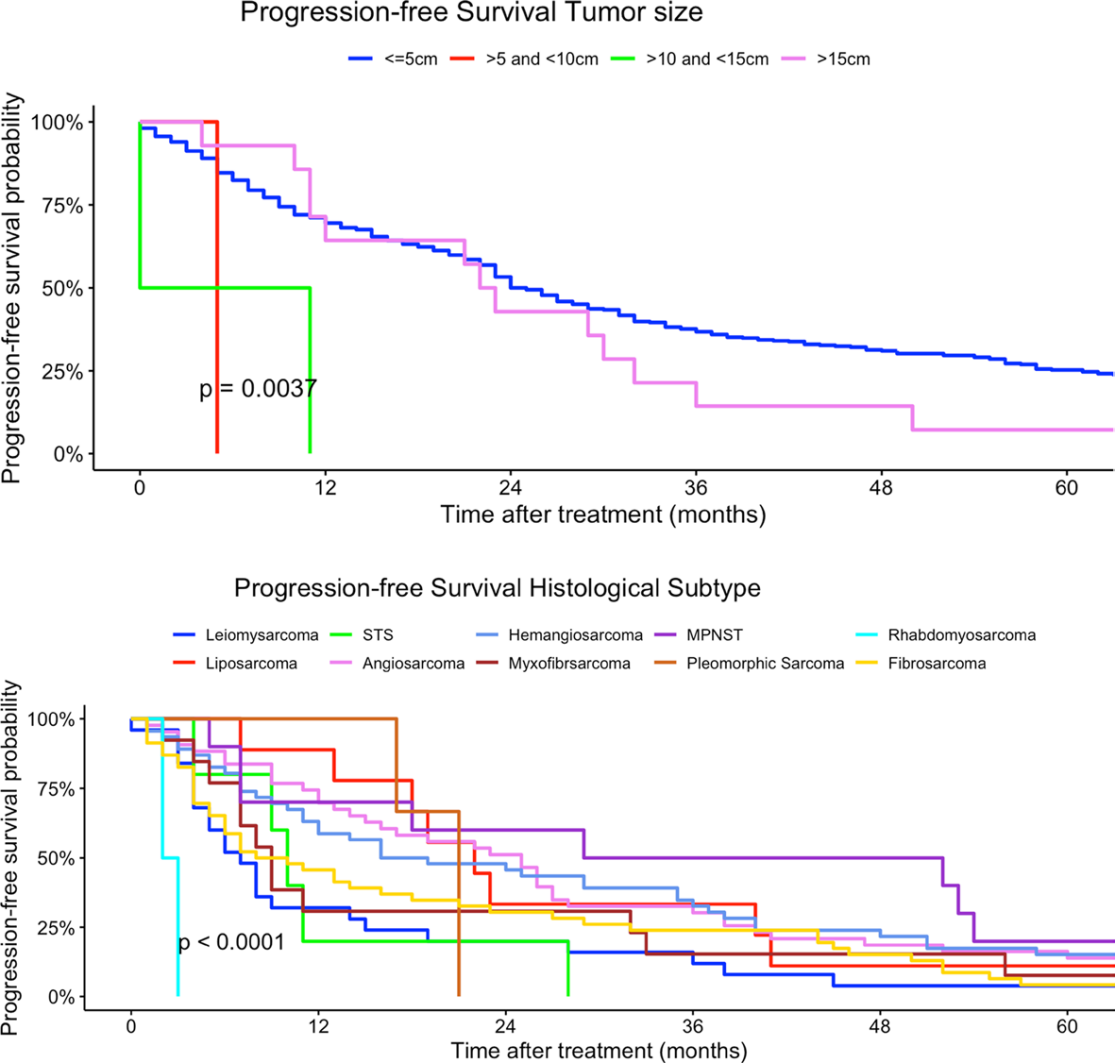
Kaplan–Meier curves of all patients (outcome: overall survival). The curve above shows tumor size. The graph below represents the association between progression-free survival and histological subtypes (STS = soft tissue sarcoma, MPNST = malignant peripheral nerve sheath tumor). All graphs were censored 60 months after treatment

Younger patients (15–29 years) had a higher risk of tumor progression compared to patients > 60 years (univariate model 15–29 years: HR 0.96, 95% CI 0.59–2.48; multivariate model 15–29 years: HR 60.5, 95% CI 2.51–1458). Tumor localization was not a prognostic factor regarding PFS in univariate and multivariate Cox regression models. Better histological grading was associated with a higher chance of non-progression in our univariate regression models (low grade: HR 0.62, 95% CI 0.42–0.89). Moreover, rhabdomyosarcoma had a higher risk in progression compared to other histological subtypes (rhabdomyosarcoma: HR 8.96, 95% CI 1.99–40.3). In comparison, liposarcoma and pleomorphic sarcoma and myxofibrosarcoma patients had a non-progression benefit related to other subtypes in our univariate regression model (liposarcoma: HR 0.45, 95% CI 0.27–0.73; leiomyosarcoma sarcoma: H 0.48, 95% CI 0.29–0.79; HR: myxofibrosarcoma: 0.29, 95% CI 0.14–0.64). Analogous to tumor localization, tumor side and tumor size had no prognostic impact on PFS in our regression models. Lower TNM-stages as well as lower UICC-stages were related to a non-progression (Table 3). Figures  7, 8, 9, 10, 11 show Kaplan–Meier curves of all included prognostic factors based on PFS.

## Discussion

The aim of this analysis was to provide an overview of prognostic factors for survival in sarcoma patients derived from data of a German sarcoma patients’ cohort provided by the public clinical cancer registry of Saxony-Anhalt. We analyzed the quality and adequacy of data of this registry to draw conclusions regarding survival and the impact of several prognostic factors in order to develop specific prediction models for survival and initiate further studies.

To our knowledge, there are only few sarcoma studies analyzing German registry data on this topic. An epidemiological cohort study of adult soft tissue sarcomas in Germany was provided from Saltus et al. in 2018. Regional German cancer registry data by the Centre for Cancer Registry Data (ZfKD) at the Robert Koch Institute (RKI) were used to describe the epidemiology of adult soft tissue sarcomas from 2003 to 2012. The most common histological categories were found to be leiomyosarcoma (19%), liposarcoma (16%) and sarcomas not otherwise specified (14%) (Saltus et al. 2018). In support of the findings by Saltus et al., in the present study, the majority of sarcoma patients were also diagnosed with leiomyosarcoma (12%) and liposarcoma (11%). The results of the study by Saltus et al. were based on data of nine German federal state registries with a rate of integrity of data sets of at least 90%. Cancer registry data from Saxony-Anhalt were not considered in their analyses (Saltus et al. 2018). As a point of criticism of the present study, one should be aware that data from a single cancer registry might lead to selection bias. Therefore, prospective analyses should include multiple cancer registries with patients from different social and ethnic backgrounds.

In 2021, Jawad et al. examined 712 patients diagnosed with primary mobile column sarcomas based on American Surveillance, Epidemiology and End Result (SEER) data. Independent predictors of survival for the entire cohort included age, grade and stage. Additionally, survival and prognostic factors varied by histological subtypes. While stage was an independent predictor of survival for every histological subtype, age, tumor size and grade were additional predictors in survival for spinal osteosarcoma, Ewing sarcoma and chondrosarcoma (Jawad et al. 2021). In future, prognostic factor analysis in sarcoma patients should be stratified by histological subtypes. To address data quality, we identified a large number of missing or unknown values especially in histological grading and T-stage. *N* = 824 (54%) patients had no documented histological grading. T-stages were missing in *n* = 691 (45%) cases (Table 1). This large number of missing values effects the interpretability of our results, especially the determination of prognostic factors. Due to the small number of available cases and the heterogeneity of entities, we decided to include also incompletely documented cases in our analyses. To create a more homogenous data set, we excluded all histological subtypes differing from soft tissue sarcoma. Future analysis should aim to add additional clinical information to the existing registry data to improve data quality.

Pan, Merchant et al. investigated risk factors including age, stage and anatomic location in synovial sarcoma patients in 2018. Overall, 154 synovial sarcoma cases were identified in the USA diagnosed between 1981 and 2014. They identified tumor size > 5.0 cm and age > 50 years as risk factors of presenting stage IV disease. For patients with early-stage disease, tumor size > 5.0 cm was also associated with worse disease-free survival (DFS) and OS. Moreover, compared extremity primary, patients with head and neck and trunk primary had lower OS (Pan und Merchant 2018). In comparison, our data set included only 23 synovial sarcoma cases diagnosed between 2005 and 2022. Analogous to the data of Pan, Merchant et al., we could also identify tumor size > 5cm as a prognostic factor in terms of OS. In our data, tumor size > 5cm could not be identified as a prognostic factor regarding PFS. Corresponding to the findings of Pan, Merchant et al., extremity sarcomas had a better OS compared to head-, trunk-, and pelvic-located sarcomas (Pan und Merchant 2018).

Further, a Chinese SEER database analysis examined survival rates of patients with osteosarcoma, chondrosarcoma, Ewing sarcoma and chordoma. Factors including age older than 40 years, higher grade, regional and distant stage, tumor in the extremities, T2-stage, bone and lung metastasis, and non-surgery were associated with poor survival of the entire cohort (Xu et al. 2021). In our regression model, older age > 60 years was associated with worse OS compared to younger aged patients. In our study, treatment groups were unequally distributed (< 40 years: *n* = 102; > 40 years: *n* = 1427). This selection bias could be compensated by propensity score matching. Due to many histological subtypes and small sample size, we did not perform propensity score matching. This method allows us to create two balanced treatment groups with identical sample sizes (Randolph et al. 2014). To focus on age-related differences, data from several cancer registries should be included in propensity score-matched risk factor analysis. Psychological adaption and recovery in young patients with sarcoma were investigated by a British group and found many individual and environmental psychological factors related to illness adaption. The authors proposed a dynamic model of psychological adaption and recovery in this population (Kosir et al. 2020).

Raedkjaer et al. examined the relation between socioeconomic factors and risk of presenting cancer-related prognostic factors in Danish soft tissue and bone sarcoma patients. In this study, patients with short education, low income, or living alone had a higher mortality. In addition, soft tissue sarcoma patients living alone had a greater risk of having a large tumor at the time of diagnosis (Raedkjaer et al. 2020). Information on socioeconomic status was not available in this cancer registry data. Future risk factor analyses should include socioeconomic data to improve sarcoma diagnostic and cancer prevention. Bedir, Abdera et al. investigated socioeconomic disparities in endometrial cancer based from the German Centre of Cancer Registry Data. Their results indicated differences in endometrial cancer survival according to socioeconomic deprivation among stage I patients (Bedir et al. 2022). Analogous analyses could be conducted for sarcoma patients based on the German Index of Socioeconomic deprivation.

A retrospective analysis of 182 patients described grade, size, histological type and age at diagnosis as prognostic for survival in extremity soft tissue sarcoma (Singer et al. 1994). Another retrospective study by Soydemir et al. revealed tumor stage, surgical method applied, radiotherapy application, RT dose and development of metastasis during follow-up as prognostic factors in extremity soft tissue sarcoma patients. Besides surgery, RT played a crucial role in multimodal treatment and increased local control rates and OS (Soydemir et al. 2020). In our study, treatment strategies were not included as prognostic factors. Further studies should consider multimodal treatment strategies as prognostic factors. Adjuvant RT, surgery and RT dose should especially be included in prognostic factor regression models.

Another important issue in sarcoma cancer research is the willingness of patients to participate in clinical studies. Schneider, Giglio et al. investigated patients’ perspectives on the burden of cancer care as well as factors that influence comfort with randomization in clinical trials. The main proportion of patients with extremity sarcoma had altruistic reasons to help future patients. Those that would decline to participate most commonly reported that participating in research would be too much of a burden (Schneider et al. 2021). Therefore, cancer registry research is a low-threshold alternative to ornate clinical trials without psychological stress for sarcoma patients.

## Conclusions

In this study, prognostic factors in sarcoma patients were evaluated based on cancer registry data. Histological grade, tumor size, nodal and distant metastasis, tumor localization and histological subtype were determined as prognostic factors in terms of survival. Tumor side, age at diagnosis and sex had no significant influence on survival. Furthermore, adding clinical information to the existing registry data would allow for more profound analysis.

## Data Availability

The datasets generated during and/or analyzed during the current study are not available from the corresponding author due to cancer registry regulations.

## Abbreviations

AJCC: American joint committee on cancer
AYA: Adolescent and young adults
CI: Confidence interval
DFS: Disease-free survival
GIST: Gastrointestinal stromal tumor
HR: Hazard ratio
ICD: International statistical classification of diseases and related health problems
OS: Overall survival
PFS: Progression-free survival
RKI: Robert koch institute
RT: Radiotherapy
SEER: Surveillance, epidemiology and end result
ZfKD: Centre for cancer registry data
